# A Comparative Genomic and Safety Assessment of Six *Lactiplantibacillus plantarum* subsp. *argentoratensis* Strains Isolated from Spontaneously Fermented Greek Wheat Sourdoughs for Potential Biotechnological Application

**DOI:** 10.3390/ijms23052487

**Published:** 2022-02-24

**Authors:** Maria K. Syrokou, Spiros Paramithiotis, Eleftherios H. Drosinos, Loulouda Bosnea, Marios Mataragas

**Affiliations:** 1 Department of Food Science and Human Nutrition, Agricultural University of Athens, 75 Iera Odos St., 11855 Athens, Greece; syrokoumargia@aua.gr (M.K.S.); sdp@aua.gr (S.P.); ehd@aua.gr (E.H.D.); 2 Department of Dairy Research, Institute of Technology of Agricultural Products, Hellenic Agricultural Organization “DIMITRA”, 3 Ethnikis Antistaseos St., 45221 Ioannina, Greece; louloudabosnea@gmail.com

**Keywords:** bioinformatics, fermentation, foods, lactic acid bacteria, starter culture, whole genome sequencing

## Abstract

The comparative genome analysis of six *Lactiplantibacillus plantarum* subsp. *argentoratensis* strains previously isolated from spontaneously fermented Greek wheat sourdoughs is presented. Genomic attributes related to food safety have been studied according to the European Food Safety Authority (EFSA) suggestions for the use of lactic acid bacteria (LAB) in the production of foods. Bioinformatic analysis revealed a complete set of genes for maltose, sucrose, glucose, and fructose fermentation; conversion of fructose to mannitol; folate and riboflavin biosynthesis; acetoin production; conversion of citrate to oxaloacetate; and the ability to produce antimicrobial compounds (plantaricins). Pathogenic factors were absent but some antibiotic resistance genes were detected. CRISPR and *cas* genes were present as well as various mobile genetic elements (MGEs) such as plasmids, prophages, and insertion sequences. The production of biogenic amines by these strains was not possible due to the absence of key genes in their genome except lysine decarboxylase associated with cadaverine; however, potential degradation of these substances was identified due to the presence of a blue copper oxidase precursor and a multicopper oxidase protein family. Finally, comparative genomics and pan-genome analysis showed genetic differences between the strains (e.g., variable *pln* locus), and it facilitated the identification of various phenotypic and probiotic-related properties.

## 1. Introduction

Lactic acid bacteria (LAB) are microorganisms that possess important technological characteristics and, therefore, are used in food fermentations as starters or even as adjunct cultures providing the desired quality and organoleptic properties and assuring the safety of the fermented products. Traditionally made fermented foods, i.e., without the addition of a commercial starter culture, constitute a significant source of isolating wild LAB strains comprising the indigenous flora of the products performing the spontaneous fermentation. Although LAB have been acknowledged as safe for human and animal consumption (Generally Recognized as Safe—GRAS by the Food and Drug Administration—FDA and Qualified Presumption of Safety—QPS by the European Food Safety Authority—EFSA) [1,2], infection is still possible—especially for immunocompromised individuals [3,4,5]. Thus, the safety assessment of microbial strains aimed to be used as potential food additives is always timely, and it is highly recommended for investigation [6].

Since they constitute the majority of the respective micro-community, LAB and yeasts play a critical role during sourdough fermentation. There are different types of sourdoughs, based on the applied fermentation and technological processes [7]. Type I sourdough, from which the six *Lactiplantibacillus plantarum* subsp. *argentoratensis* strains under study were isolated, is characterized by the dominance of *Fructilactobacillus sanfranciscensis*, *Levilactobacillus brevis*, *Limosilactobacillus fermentum*, *L. plantarum*, and *Companilactobacillus paralimentarius* [8]. In the latter work, Greek wheat sourdoughs collected from various geographical regions were analyzed by using culture-dependent and culture-independent molecular techniques. These six strains, initially identified as *L. plantarum* by 16S rRNA gene sequencing, were further selected for whole-genome sequencing based on their phenotypic and technological properties [9]. Taxonomic classification of the strains based on the sequenced genome revealed that the isolated colonies belong to the subspecies *argentoratensis*. Sequenced genomes of *L. plantarum* subsp. *argentoratensis* are not readily available, especially strains isolated from sourdoughs.

Nowadays, as the sequencing technology has advanced and the cost of genome sequencing has decreased, the use of bacterial whole-genome sequencing as a diagnostic tool is more feasible than a few years ago. When a genome is available completed or as a draft of high quality, its bioinformatic analysis offers an unprecedented way of investigating the biotechnological potential and safety of the isolated sequenced strains [10,11]. Recently, Carpi et al. (2022) [12] performed a detailed pan-genome analysis of *L. plantarum* using 127 complete genomes. Another three related works have been conducted using the draft genomes of *L. plantarum* strains [13,14,15]. However, the main limitation of those studies is that acquired antibiotic resistance (AR) genes were annotated using only the Comprehensive Antibiotic Resistance Database (CARD) and/or ResFinder databases, resulting in many cases without any hits or in only one hit. In those works, the authors concluded that AR genes were not detected in the *L. plantarum* genomes. Chokesajjawatee et al. (2020) [6] mentioned that the limitation of the AR genes search using the CARD and ResFinder databases for non-pathogenic bacteria should be marked because both databases are mainly focused on the AR genes of pathogenic bacteria and those of the non-pathogenic are usually not included, resulting in their inability to identify the AR genes in *L. plantarum* genomes at the default. On the contrary, the search of the AR genes using the Kyoto Encyclopedia of Genes and Genomes (KEGG) database proved to be efficient for this purpose.

Consequently, in this study the genomes of six *L. plantarum* subsp. *argentoratensis* strains, isolated from spontaneously fermented Greek wheat sourdoughs, were bioinformatically analyzed and compared in relation to their biotechnological features and their safety for potential application to foods. In addition, there is no report on the bioinformatic analysis of the *L. plantarum* subsp. *argentoratensis* genomes, isolated from spontaneously fermented Greek sourdoughs, and from sourdoughs in general, considering the very limiting number of the available genomes of this subspecies in the NCBI database. The bioinformatic analysis of the genome of the *L. plantarum* subsp. *argentoratensis* strains performed in the current work will provide insights regarding their genomic and functional features and their potential use as a food additive.

## 2. Results and Discussion

### 2.1. Strain Relatedness

The complete (100%) sequence of the 16S rRNA gene was extracted from the sequenced genome of all strains using the ContEst16S webtool and its length was 1567 bp. It should be noted, however, that although the genomes were of high quality (number of contigs was far below the limit of 200, the sequence length of each contig was ≥500 bp, and the genome coverage was much higher than the threshold of 60–80× and the recommended 100×) they were not closed because it is well known that the genome of lactic acid bacteria contains repeat regions and other mobile elements that make it difficult to finish the genome using only short read sequences [16,17].

**Figure 1:**
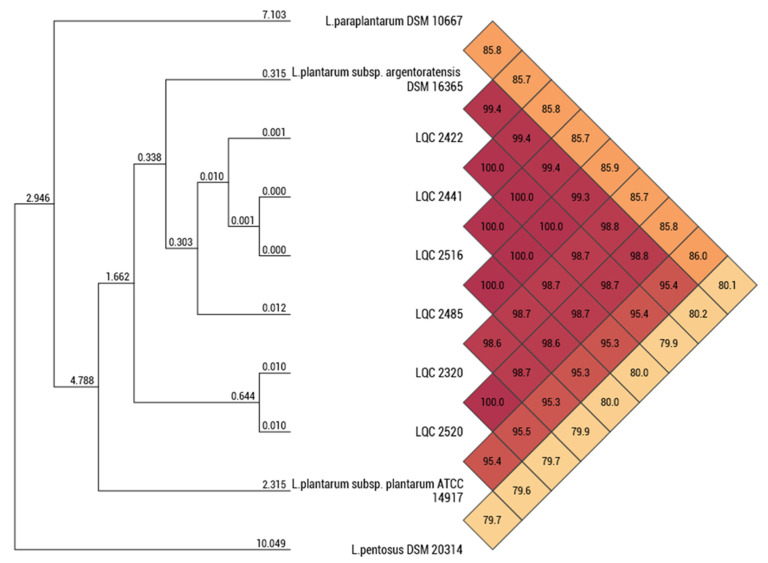
Calculated ANI values showing the relatedness between the sequenced genomes of the strains isolated from traditional Greek wheat sourdoughs (LQC 2422, LQC 2441, LQC 2516, LQC 2485, LQC 2320, and LQC 2520) and the type strains restricted to those displayed a very high 16S rRNA gene similarity (≥99.80%).

**Figure 2:**
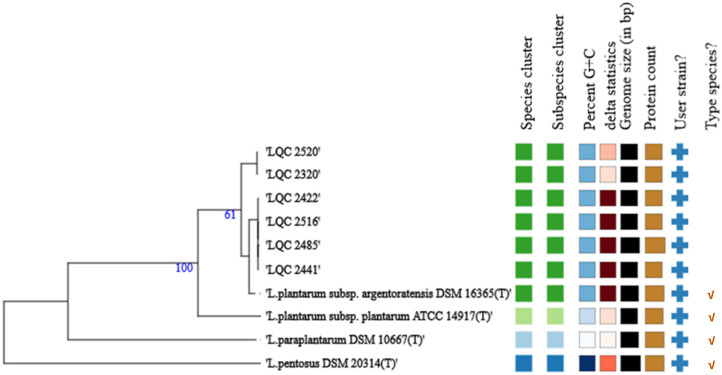
A bootstrapped genome-based phylogenetic relationship of the strains isolated from traditional Greek wheat sourdoughs (LQC 2422, LQC 2441, LQC 2516, LQC 2485, LQC 2320, and LQC 2520) and the type strains restricted to those displayed a very high 16S rRNA gene similarity (≥99.80%). The numbers above branches are pseudo-bootstrap support values > 60% from 100 replications. Matrix on the right (columns from left to right), dDDH species (>70%) and subspecies (>79%) cluster, strains with the same color belong to the same species or subspecies group; GC content (%), min 43.9 (*L. paraplantarum* DSM 10667^T^) and max 46.3 (*L. pentosus* DSM 20314^T^), strains with the same color have similar GC content (e.g., strains of this study and *L. plantarum* subsp. *argentoratensis* DSM 16365^T^ have a GC content of 45.0–45.1); delta (*δ*) values (<1) showing the tree-likeness of the data set, if a strain has an exceptionally high value this indicates that this genome should be removed because it negatively affects phylogenetic inference; Genome size in Mb, min 3.13 (LQC 2422) and max 3.67 (*L. pentosus* DSM 20314^T^); Protein content, min 2958 (LQC 2422) and max 3310 (*L. pentosus* DSM 20314^T^); Strains provided by the user (cross symbol); and Type species (tick symbol).

The 16S-based ID platform of the EzBioCloud server revealed a high sequence similarity with the following type strains: *L. pentosus* DSM 20314^T^ (99.87%), *L. paraplantarum* DSM 10667^T^ (99.80%), *L. plantarum* subsp. *argentoratensis* DSM 16365^T^ (99.80%), and *L. plantarum* subsp. *plantarum* ATCC 14917^T^ (99.80%). The results from the 16S-based ID showed additional strains with sequence similarity above the threshold of 98.70% (*L. fabifermentans* DSM 21115, 99.19%; *L. daowaiensis* 203-3, 99.17%; *L. pingfangensis* 382-1, 99.16%; *L. garii* FI11369, 99.10%; *L. nangangensis* 381-7, 99.07%; *L. daoliensis* 116-1A, 99.03%; *L. herbarum* TCF032-E4, 98.99%, *L. xiangfangensis* LMG 26013, 98.92%; and *L. plajomi* NB53, 98.79%) but these were not selected further for ANI comparisons because the results obtained from the TYGS webtool indicated very poor pairwise dDDH values (<23%) between these strains and the strains of the current study. The calculated ANI values between the genomes of this work and the closely related type strains (16S rRNA gene sequence similarity ≥99.80%) are displayed in Figure 1. Based on the threshold ANI value of 95–96% and those calculated between the genomes, the six microbial strains isolated from traditional Greek wheat sourdoughs were identified as *L. plantarum* subsp. *argentoratensis*. In addition, the results obtained from the TYGS platform on the pairwise comparisons of user genomes and type-strain genomes, restricted to the above-mentioned four type strains, confirmed that these strains belong to the species of *L. plantarum* subsp. *argentoratensis* (dDDH value >79%) (Table 1, Figure 2). According to the Qualified Presumption of Safety (QPS) list of EFSA [1] the species *L. plantarum* has received QPS status.

Some strains presented a high nucleotide sequence identity approximating one hundred percent, although they had been isolated from geographically distinct sites. This could be explained by the fact that orthoANI calculates only the similarity of orthologous fragments. For example, the strains LQC 2422 and LQC 2516 presented genetic differences, such as the length and the organization of the *pln* loci encoding for plantaricins, which discriminated the two strains (see Section 2.3 “Safety assessment”). A high similarity has been also reported between *L. plantarum* strains of different origin [12].

### 2.2. Phenotypic Characterization and Pan-Genome Analysis

#### 2.2.1. Phenotypic Properties

**Figure 3:**
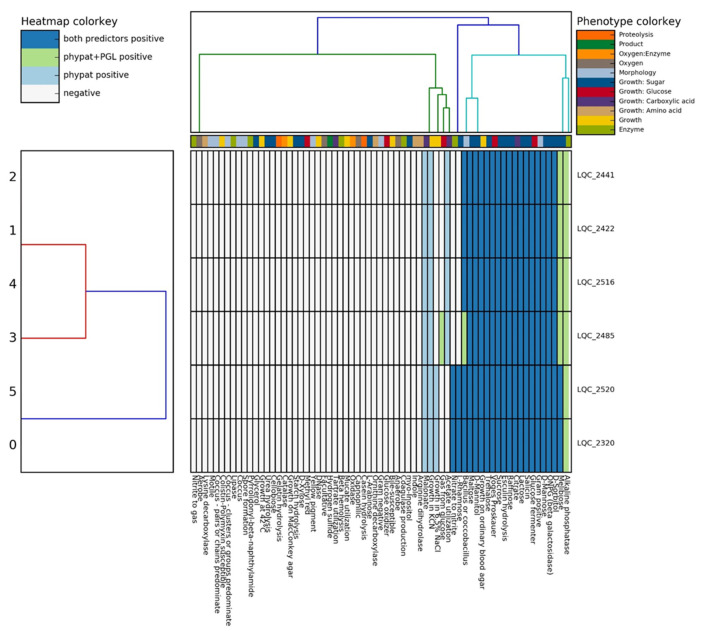
Heatmap of the phenotypic features of the strains isolated from traditional Greek wheat sourdoughs (LQC 2422, LQC 2441, LQC 2516, LQC 2485, LQC 2320, and LQC 2520) as predicted by the Traitar webtool.

Based on the phenotype prediction (Figure 3), the six strains were: Gram-positive, catalase-negative, non-motile and non-sporeforming bacilli; unable to grow at 42 °C and produce NH_3_ from arginine; and negative to indole production, H_2_S formation, urease activity, and oxidase test. These properties are very common in the genus of *Lactobacillus* [18]. Regarding the sugar fermentation profile of the isolates, these showed a positive pattern for maltose, melibiose, sorbitol, mannose, glucose, salicin, lactose, raffinose, esculin, sucrose, trehalose, and mannitol, but they were negative for cellobiose, xylose, myo-inositol, and arabinose fermentation (Figure 3). However, genome annotation showed that the strains harbored the respective genes (EC 2.7.1.205 and EC 3.2.1.86) for the utilization of cellobiose. These predictions are in line with the experimental phenotypic tests conducted on the isolates by Syrokou et al. (2020) [8] who found that the *L. plantarum* subsp. *argentoratensis* strains can ferment a wide range of carbohydrates, including the cellobiose. Moreover, genome annotation analysis showed that the strains were able to ferment fructose and convert fructose to mannitol, a well-known low-calorie sweetener. Also, the strains LQC 2320 and 2520 were able to catabolize rhamnose and reduce nitrate to nitrite. The latter is mediated by the nitrate reductase which under certain circumstances may lead to nitric oxide (NO) synthesis, a property of great importance during fermented sausage manufacturing. The *L. plantarum* AJ2 strain isolated from naturally fermented sausage possessed the ability to reduce the residual levels of nitrite and nitrate [19].

Alkaline phosphatase production by all strains and gas (CO_2_) production from glucose by the LQC 2485 strain was projected by the phypat + PGL predictor only, while the phypat showed a positive result toward acetate utilization (strains LQC 2441, LQC 2422, LQC 2516, and LQC 2485) and growth in 6.5% NaCl (strains LQC 2520 and LQC 2320) (Figure 3). However, positive results predicted only by the phypat should be evaluated experimentally. Weimann et al. (2016) [20] noted that the phypat predictor tends to give more false-positive results when draft genomes are used. Alkaline phosphatase production by *L. plantarum* has been linked with the increased degradation rate of the organophosphorus pesticides present in wheat dough or skimmed milk [21,22]. Other important phenotypic properties predicted by Traitar were the strains’ ability to utilize the citrate as a carbon source, which may lead to the formation of aroma precursor compounds; the strains’ ability to produce the enzyme beta-galactosidase (ONPG); and the positive reaction to the Voges–Proskauer (VP) test. Beta-galactosidases belong to glycoside hydrolase (GH) groups. Genome annotation revealed that all strains had the GH2 group of beta-galactosidase and especially the LacLM type encoded by two genes, *lacL* and *lacM* (EC 3.2.1.23) (the other type is the LacZ, encoded by the *lacZ* gene). While *L. plantarum* predominantly possess the LacLM type [23], the LacZ type is found in other LAB [24]. The enzyme hydrolyzes lactose and, therefore, it is used in the dairy industry for health-related applications like the production of lactose-free products or prebiotic galactooligosaccharides (GOS). The addition of beta-galactosidase-producing lactobacilli as probiotics to dairy products can assist lactose-intolerant individuals. The addition of GOS stimulates the growth of probiotics and regulates the gut microflora [25]. The positive VP test, which was negatively correlated with the methyl red test (the MR test was predicted as negative) (Figure 3), indicates the presence of acetoin, which is the precursor of 2,3 butanediol and other aromatic compounds. Thus, the strains do not use the mixed acid fermentation pathway, i.e., production of several organic acids from glucose utilization, but they use the metabolic pathway that leads to the formation of 2,3 butanediol, in addition to lactate production. Thakur et al. (2017) [18] found that a *L. plantarum* strain isolated from pickle samples displayed the opposite phenotype, i.e., MR+ and VP−. Usually, a bacterial culture is positive to only one pathway, either VP+ or MR+. Indeed, the results on the genome annotation of *L. plantarum* subsp. *argentoratensis* confirmed the presence of a complete set of genes for acetoin production and conversion of citrate to oxaloacetate. The latter coincided with the predictions made by Traitar. Finally, 15 GHs (glycoside hydrolases) and 9 GTs (glycosyltransferases) families contained in the CAZymes database were spotted, indicating a high variability in GH- and GT-encoding.

#### 2.2.2. Pan-Genome Analysis and Annotation

**Figure 4:**
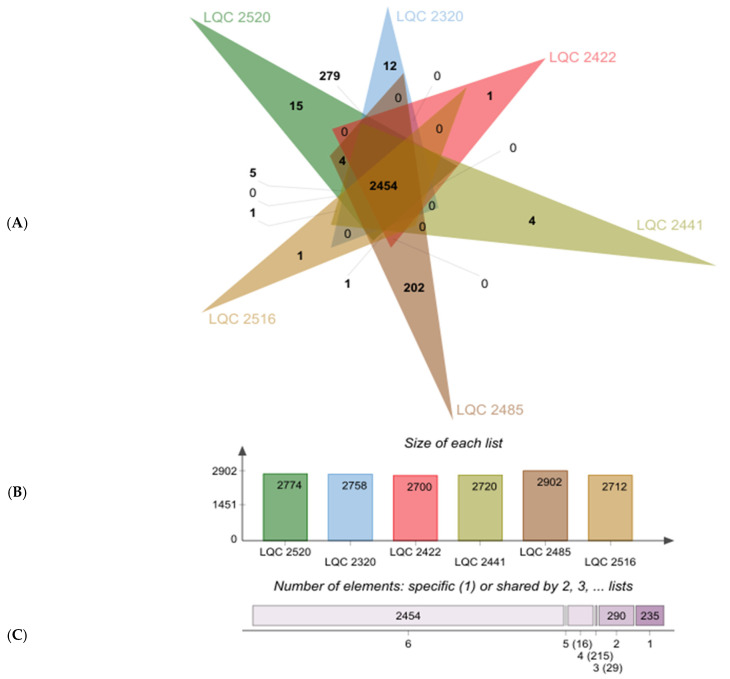
Venn diagram (**A**) showing the number of genes located in the core-, accessory- and unique genome of each *L. plantarum* subsp. *argentoratensis* strain. The plots below the Venn diagram show the total number of genes in each genome of the strains (**B**) and the total number of genes shared by the respective number of genomes (**C**). Venn diagram was constructed using the jvenn webtool [32].

According to Heaps’ law [26], the curve fitting of the pan-genome resulted in an estimated a value of 0.10 (a < 1) which means that when a new genome is added to the pan-genome, new genes are added as well increasing in this way its size (open pan-genome). The increase of pan-genome by 0.10 implies potential evolution-related changes in the *L. plantarum* subsp. *argentoratensis* genomes (horizontal gene transfer—HGT, gene gain or gene loss) to cope with the adaptation to various environmental conditions. The pan-genome of the six strains comprised of 3239 genes in total, from which 2454 genes were in the core-genome, 550 genes in the accessory-/unique-genome, and 235 genes were unique (Figure 4). The strain LQC 2485 harbored the highest number of unique genes (202) compared to the other strains. Hence, gene exchange activities associated with potentially different approaches of adaptation and response to environmental conditions are happening at the highest rate in this specific strain. For instance, the genome annotation performed revealed the presence of a multiple-sugar metabolism (*msm*) gene cluster analogous to the described system found in *Streptococcus mutans* [27,28]. This multiple-sugar ABC transporter were comprised of two permease proteins (*msmG* and *msmF*) and a substrate-binding protein (*msmE*). Another feature found in the unique-genome of the LQC 2485 strain is the presence of a pyruvate dehydrogenase (quinone) (EC 1.2.5.1), which stimulates the conversion of pyruvate to acetate.

**Figure 5:**
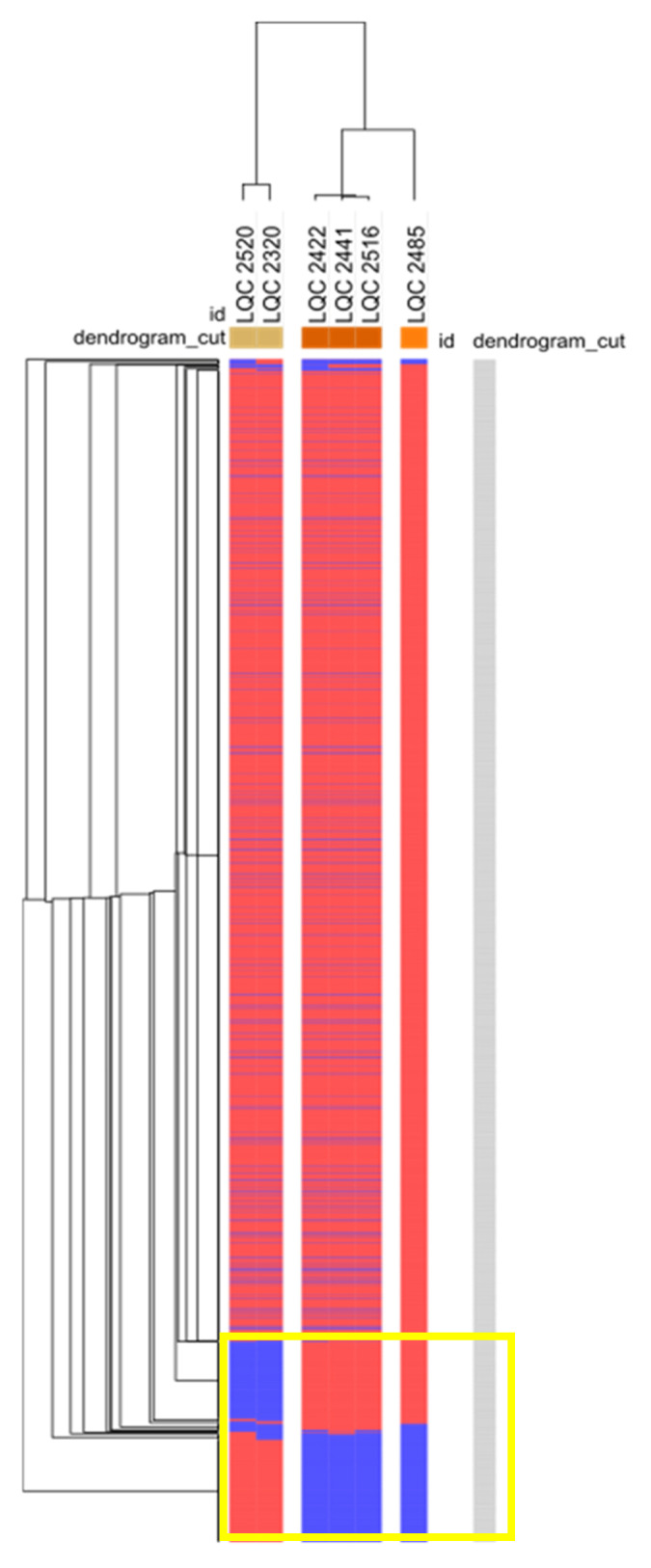
Heat-map of the genes presence (red) and absence (blue) of the six *L. plantarum* subsp. *argentoratensis* genomes. Yellow box marks the difference in the genes presence/absence between the two microbial clusters.

The hierarchical clustering of the gene matrix (presence/absence) (Figure 5), acquired during the pan-genome analysis of the *L. plantarum* subsp. *argentoratensis* genomes, was like the clustering obtained based on the WGS (Figure 2). However, the LQC 2485 was quite distinct from the other two groups probably due to the high number of unique genes harbored by this strain. The LQC 2320 and LQC 2520 isolates formed a separate group from the rest of the strains (LQC 2422, LQC 2441, and LQC 2516) attributed to the presence/absence of a group of genes in each microbial cluster (Figure 5). Thus, HGT, gene gain, or gene loss phenomena potentially have occurred between the *L. plantarum* subsp. *argentoratensis* strains and other microbial clades. Indeed, the LQC 2320 and LQC 2520 strains possessed a genes cluster related to the reduction of nitrate to nitrite encountered in *Staphylococcus carnosus* [29] (nitrate/nitrite transporter, NarT; oxygen-sensing two component system sensor histidine kinase, NreBC; and respiratory nitrate reductase alpha, beta, gamma, and delta chain; *narGHIJ*, EC 1.7.99.4), and the utilization of L-rhamnose (transcriptional regulator of rhamnose utilization, AraC family, L-rhamnose permease, RhaY, major facilitator superfamily—MFS; L-rhamnose mutarotase, RhaM, EC 5.1.3.32; L-rhamnose isomerase, RhaA, EC 5.3.1.14; L-rhamnulose kinase, RhaB, EC 2.7.1.5; and L-rhamnulose-1-phosphate aldolase, RhaD, EC 4.1.2.19); both features were predicted by the Traitar and annotated by the respective web-services. On the other hand, the LQC 2422, LQC 2441, LQC 2485, and LQC 2516 strains harbored the genes cluster associated with the maltodextrin-specific ABC transporter, i.e., the ATP-binding protein MdxE, the membrane-spanning components MdxF and MdxG, and the energizing ATPase MsmX [30], and the utilization of glycerate (phosphotransferase System—PTS, 2-O-alpha-mannosyl-D-glycerate-specific IIA, IIB, and IIC component, EC 2.7.1.195; glycerate kinase, EC 2.7.1.31; and mannosylglycerate hydrolase, EC 3.2.1.170). Annotation showed the presence of 2-dehydro-3-deoxy-D-gluconate-5-dehydrogenase (EC 1.1.1.127) and 2-deoxy-D-gluconate-3-dehydrogenase (EC 1.1.1.125) in the genome of the above four strains where the resulting compound enters the pentose phosphate pathway. Finally, all strains harbored a PTS-system, fructose-specific IIA, IIB, and IIC component (EC 2.7.1.202) and a PTS-system, mannose-specific IIA, IIB, IIC, and IID component (EC 2.7.1.191), but only these four strains had a PTS-system, fructose- and mannose- inducible IIA, IIB, IIC, IID, and IIE component. This phosphoenolpyruvate: fructose phosphotransferase system constitutes an alternative way of entering mannose into *Streptococcus salivarius* [31].

**Figure 6:**
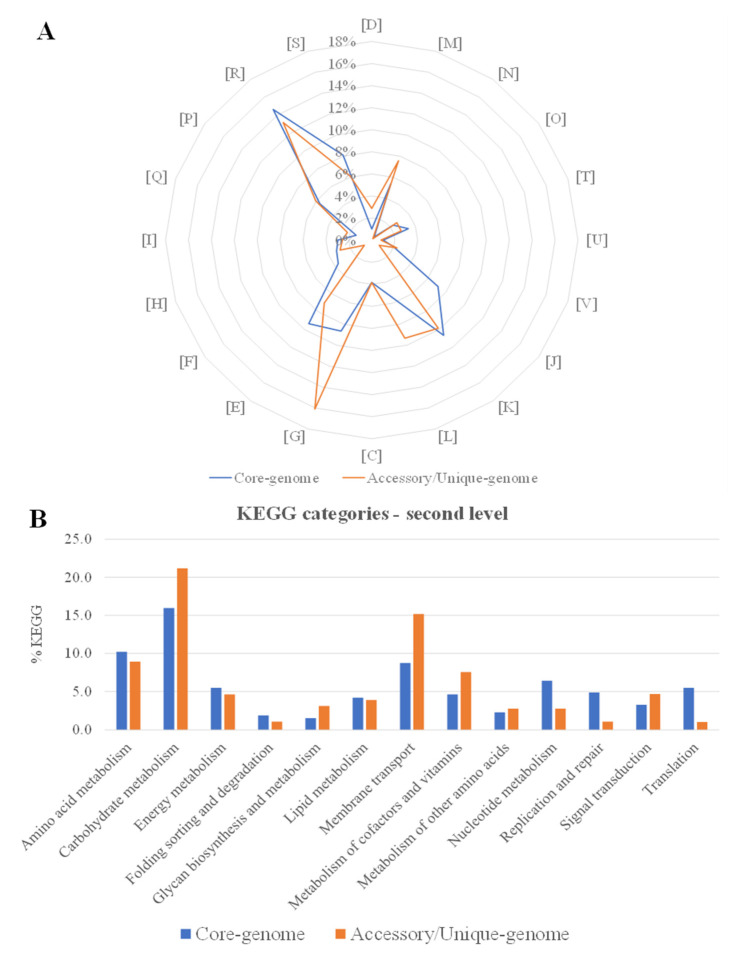
Plots displaying the distribution of the COG (**A**) and KEGG (**B**) functional categories in the core- and accessory-/unique-genome of the *L. plantarum* subsp. *argentoratensis* strains.

Gene assignment to the different COG functional groups depicted that the genes of translation, ribosomal structure and biogenesis (J), amino acid transport and metabolism (E), and nucleotide transport and metabolism (F) were enhanced in the core-genome of the *L. plantarum* subsp. *argentoratensis* species (Figure 6A), i.e., genes implicated in microbial housekeeping processes [33]. Genes related to cell wall/membrane/envelop biogenesis (M), DNA replication, recombination and repair (L), and carbohydrate transport and metabolism (G) were enhanced in the accessory-/unique-genome, i.e., genes implicated in energy metabolism and DNA repair (Figure 6A) [33]. Moreover, the genes involved in carbohydrate metabolism and membrane transport were the most abundant in the *L. plantarum* subsp. *argentoratensis* strains confirming the capacity to metabolize different carbohydrates, a feature which could be strain-dependent (higher abundance of genes in the accessory-/unique-genome compared to the core-genome) (Figure 6B). For example, as discussed above, the Traitar platform predicted that the LQC 2320 and LQC 2520 strains ferment L-rhamnose, but the others do not have this ability. Another observation worth-mentioning is the relatively low gene abundance in the lipid transport and metabolism category (Figure 6), suggesting that the strains’ lipolytic activity is not high. Indeed, experimental results obtained by Syrokou et al. (2020) [8] on the lipolytic activity of the strains under study confirm: strains LQC 2422, LQC 2441 and LQC 2485 exhibited no lipolytic activity, strains LQC 2520 and LQC 2320 showed moderate activity (22.75 AU/mL and 37.25 AU/mL, respectively), and strain LQC 2516 showed low activity (14.50 AU/mL).

All strains displayed the presence of a complete set of genes for riboflavin and folate biosynthesis. Similarly, *L. plantarum* strains isolated from quinoa sourdough [34] or a traditional maize-based fermented beverage (chicha) [16] had this ability as well. All *L. plantarum* subsp. *argentoratensis* strains seem to produce the foldase protein PrsA encoded by the *prtM* gene, which is responsible for the cleavage of proteins and the subsequent utilization of the large polypeptides by the microbial cells. The Opp (*oppA*, *oppA2*, *oppBCDF*) and Dtp (*dtpT*) gene clusters identified in their genomes widen the peptide uptake mechanisms. These peptides are subsequently hydrolyzed by various peptidases and proteases such as oligoendopeptidases (*pepE*, *pepF*, *pepF2*, *pepO*, *pepC*, and *pepN*), proline-specific peptidases (*pepX*, *pepI*, *pepQ*, and *pip*), di- and tri- peptidases (*pepV*, *pepT*, and *pipD*), and other enzymes (*map*—methionine aminopeptidase; *mccF*—LD-carboxypeptidase; *est*—serine aminopeptidase; *ydcK*—*SprT* family; *ddpX*—hydrolysis of the D-alanyl-D-alanine dipeptide; *vanY*—D-alanyl-D-alanine carboxypeptidase; and *ctpA*, *dacA*, *amd*, *gluP*, *lepB*, *trpG*, *ytzB*, *ymfH*, *ymfF*, *glbL*, *htpX*, *yvpB*, peptidases belonging to different families).

Various probiotic-related genes were identified in the genomes of the six strains suggesting their potential probiotic properties [35,36]. These genes were associated with stress response and immunomodulation (*uspA*, *dltA*, *dltB*, *dltC*, *dltD*), salt-stress (nitrate/sulfonate/bicarbonate ABC transporter), acid-tolerance (*gadB*), DNA protection (ImpB/MucB/SamB and Dps family proteins, *clpB*, *clpC*, *clpL*, *msrB*, *luxS*), and adhesion ability (mucin-binding protein—MucBP domain, collagen binding domain protein which encodes an adhesion potentially related to colonization and competition against pathogens, LPxTG-motif cell wall anchor domain protein, *fbpA*, and *groS*). Adhesion could also be related to exopolysaccharides (EPS) or surface proteins. However, no *cps* cluster (*cps1*, *cps2*, *cps3*, *cps4*) was recognized; only the *cps2J* gene (accessory-genome) from cluster 2 and the *cps4J* gene (core-genome) from cluster 4. On the contrary, the surface protein Ef-Tu was identified, which acts as an adhesion factor. Such probiotic-related properties have been found in other *L. plantarum* strains such as ZJ316 [35] and microbes isolated from Indian fermented foods [36]. Finally, choloylglycine hydrolase family proteins (*pva1*, *pva2*, *pva3*, *cbh*; EC 3.5.1.24) referred to as bile salt hydrolases (BSH) were detected indicating bile adaptation. This observation confirms the prediction made by the Traitar platform on bile sensitivity of the strains which were negative. Although, bile salt resistance has been used as a criterion, among others, for the selection of a strain as probiotic, this activity could be potentially damaging to the human host and, therefore, the use of the BSH feature as a desirable property of the probiotic microorganism is controversial [6,37].

### 2.3. Safety Assessment

It is well known that bacteria may produce various biogenic amines (BAs) in foods, leading to health side-effects [38]. Therefore, the candidate microbial cultures should be previously assessed for their ability to produce such compounds before their application to fermented foods as starters because there is the risk of biogenic amines accumulation owing to LAB intensive metabolic activity. To identify the presence of the most important BAs-related genes in the genome of the six *L. plantarum* subsp. *argentoratensis* strains, the KEGG database through the egg-NOG mapper results was searched. Genes associated with tyramine, phenylethylamine, putrescine, spermidine, spermine, histamine, agmatine, and tryptamine production were not located in any of the queried genomes. The *arcB* gene (ornithine carbamoyl transferase) was present in the genome of the strains but the rest of the gene cluster (i.e., *arcA*, *arcC*, and *arcD*) implicated in the ornithine conversion (decarboxylation) to putrescine was lacking. Thus, the six strains can be considered as putrescine-negative producers. On the other hand, the *cadA* gene (lysine decarboxylase), which is associated with the production of cadaverine (CAD), was found in all strains under study. The *cadBC* genes involved in the regulation and transportation system of the CAD were not found. Nevertheless, the homologous *pot* gene cluster (*potABCD*) was available in all genomes, encoding for the putrescine/ornithine antiporter system, which can be used instead of the *cadB* antiporter system of CAD/lysine [38]. However, the concentration of CAD should be experimentally determined in order to evaluate the true risk of CAD accumulation. Moreover, the strains harbored some genetic properties related to BA degradation, such as the presence of a blue copper oxidase (CueO) precursor and a multicopper oxidase protein family.

The *L. plantarum* subsp. *argentoratensis* strains are able to produce D-lactate because of the presence of D-lactate dehydrogenase and LarA which is implicated in lactate racemization. Hence, the consumption of foods containing LAB with D-lactate producing capabilities may cause problems for people with a high risk of D-lactate acidosis [6], and a precaution is needed. The screening against the CARD and PathogenFinder databases resulted in no identification of antibiotic resistance- or virulence- related genes, respectively. The microorganisms were not predicted as human pathogens since the strains’ probability of being a human pathogen was low, ranging from 4.6 to 8.4%. Chokesajjawatee et al. (2020) [6] noted that one limitation of the CARD database is its focus on the AMR of pathogenic microorganisms, while the AMR-related elements of non-pathogenic microbes are not considered; and, therefore, the search for any antibiotic resistance property against the KEGG database is recommended. Indeed, the results showed seven AMR-related elements (Table 2). However, further experimental investigation on these large families of antibiotics is needed because the possession of the respective genes does not necessarily warrant their resistance as well. For instance, Chokesajjawatee et al. (2020) [6] found that the presence of a macrolide resistance gene *msrA* and two beta-lactamase genes (*penP* spotted on two different locations) in the genome of *L. plantarum* BCC 9546 did not warrant resistance to erythromycin and ampicillin, respectively. According to the authors, this could be attributed to several factors such as the gene expression level and the substrate specificity of the expressed product. Similarly, the identification of hemolysin III protein, the product of the *hly* gene, is another example [6]. All of these undesirable genes (Table 2) located in the genome of the six strains are commonly encountered in several *L. plantarum* strains including the reference strain WCFS1 and various probiotics (JDM1, ST-III, and 299V) [6]. Such observations are suggesting the widespread presence of the related genes within the species without jeopardizing, however, their safety level compared to the existing starter/probiotic cultures. The isolated bacteria were bioinformatically examined for the production of antimicrobial drugs, particularly those deemed as clinically important. Based on the performed KEGG database search, no strain displayed the ability to produce any of the concerned antimicrobials.

Another significant factor for the safety assessment of the candidate starters/probiotics is their genome stability. The presence of various mobile genetic elements (MGEs) such as plasmids, insertion sequences (ISs), and prophages may act as vehicles of the horizontal transfer of virulence and/or AMR genes. The conducted analysis returned no virulence or AMR genes within the intact prophage regions (all strains contained two complete phage regions except LQC 2485), plasmids (at least one plasmid was identified; rep28 and repUS73 in LQC 2441, LQC 2485, LQC 2422, and LQC 2516; rep28 in LQC 2320 and LQC 2520), and IS (at least one IS was identified except of strain LQC 2485 in which no IS was located; ISLsa1—IS30 family in LQC 2441 and LQC 2422; ISP1—ISL3 family in LQC 2520; ISP2—IS1182 family and ISLsa1—IS30 family in LQC 2516; ISP1—ISL3 family and ISLpl1—IS30 family in LQC 2320). Similar MGEs have been frequently found in other *L. plantarum* strains [6,16,39] that pose no safety risk. The *L. plantarum* subsp. *argentoratensis* genomes contained two complete phage regions (intact) and some phage remnants (incomplete). Briefly, LQC 2320 and LQC 2520 had two intact regions of 26.2 Kb (total proteins, 29; phage proteins, 27; hypothetical proteins, 2; tRNA, 0; phage-related keywords found in protein names in the region, terminase, portal, head, capsid, tail, plate, and lysin; putative phage attachment site, no; most common phage, PHAGE_Lactob_jlb1; GC, 43.84%) and 52 Kb (total proteins, 71; phage proteins, 47; hypothetical proteins, 24; tRNA, 6; phage-related keywords found in protein names in the region, lysin, tail, head, portal, terminase, and integrase; putative phage attachment site, yes; most common phage, PHAGE_Lactob_Lj965; GC, 41.63%). The LQC 2485 possessed one intact region of 106.7 Kb (total proteins, 120; phage proteins, 91; hypothetical proteins, 29; tRNA, 6; phage-related keywords found in protein names in the region, integrase, lysin, terminase, portal, head, tail, protease, and capsid; putative phage attachment site, yes; most common phage, PHAGE_Lactob_Sha1; GC, 42.18%). Finally, PHASTER identified two intact regions for the strains LCQ 2422, LQC 2441, and LQC 2516 of 50.4 Kb (total proteins, 51; phage proteins, 45; hypothetical proteins, 6; tRNA, 2; phage-related keywords found in protein names in the region, integrase, tail, terminase, head, portal, protease, capsid, and lysin; putative phage attachment site, yes; most common phage, PHAGE_Lactob_Sha1; GC, 42.65%) and 51.3 Kb (total proteins, 65; phage proteins, 46; hypothetical proteins, 19; tRNA, 5; phage-related keywords found in protein names in the region, lysin, tail, head, portal, terminase, and integrase; putative phage attachment site, yes; most common phage, PHAGE_Lactob_Lj965; GC, 41.69%).

The results from the screening of CRISPR sequences are presented in Table 3. Each genome had four Class 1 CRISPR systems, except strain LQC 2485 that had five with different evidence levels (1, 3 or 4). In all cases, only two CRISPR regions of evidence level 4 had one *cas* gene cluster per CRISPR array in vicinity, which provided immunity against foreign DNA. The TypeIE CRISPR*cas* system has been associated with the discrimination of self from non-self DNA [40]. This could be a means to avoid acquiring virulence and/or AMR genes through the horizontal gene transfer phenomenon [41]. The remaining CRISPR regions were of low evidence level or without *cas* genes nearby; and, therefore, these regions were not a real or a functional CRISPR array, respectively [16,42].

**Figure 7:**
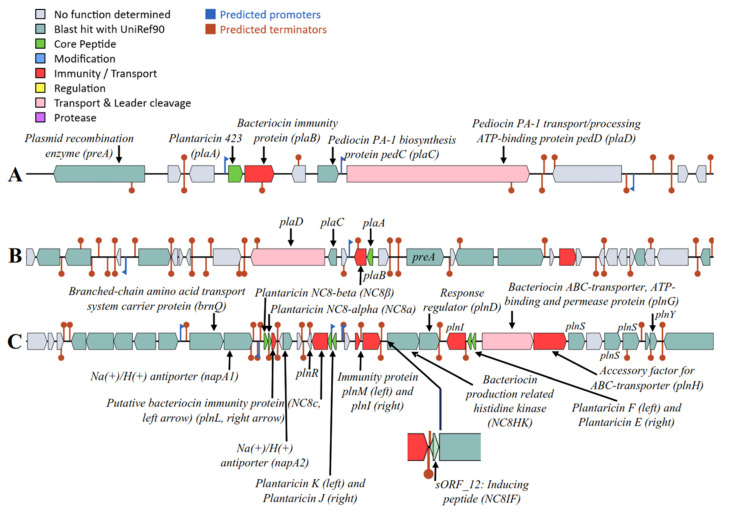
Organization of the *pln* loci of the six *L. plantarum* subsp. *argentoratensis* strains. (**A**) LQC 2485, LQC2441, and LQC 2422, (**B**) LQC 2516, and (**C**) LQC 2320 and LQC 2520.

The screening of the six genomes with the BAGEL4 platform indicated a variable *pln* locus encoding for plantaricins and spanning in a region of 8.2 kb (LQC 2485, LQC 2441, and LQC 2422), 20.2 kb (LQC 2516), and 28.9 kb (LQC 2320 and LQC 2520) (Figure 7). The organization of the *pln* locus for the strains LQC 2485, LQC 2441, LQC 2422, and LQC 2516 resembled that of the *L. plantarum* 423 strain consisting of four genes, *plaABCD* [43,44]. Plantaricin 423 is a small pediocin-like plasmid-encoded protein that belongs to the class IIa bacteriocins with a double-glycine leader peptide and strong antilisterial activity. The strains LQC 2320 and LQC 2520 harbored genes encoding for three class IIb bacteriocins, *plnJK* (two-peptide plantaricin), *plnEF* (two-peptide plantaricin), and *NC8βα* (inducible plantaricin) [45]. The structure of the *pln* operon in these strains was found to be like that of *L. plantarum* strain NC8 [46,47]. More accurately, the presence of the operons *plnJKLR*, *plnEFI*, and *plnC8αβc* was verified; while, in the case of the transport operon, the presence of only three genes—*plnG*, *plnH*, and *plnS*—was evident, followed by *plnY*, which was absent in *L. plantarum* NC8. As far as the regulatory operon was concerned, the presence of three genes—*plNC8-IF*, *plNC8-HK*, and *plnD—*encoding an induction pheromone, a histidine protein kinase, and a response regulator, respectively, was observed. The presence of *plnM* was also reported. Regarding the *plNC8-IF*, it was indicated as a small open reading frame (ORF) labelled by BAGEL4 as sORF_12 (Figure 7C). Its sequence was blasted using the BLAST protein tool with the default settings and the results returned a plantaricin precursor peptide, induction factor (98%, *L. plantarum*).

## 3. Materials and Methods

### 3.1. Microbial Strains

The bacterial strains LQC 2320, LQC 2422, LQC 2441, LQC 2485, LQC 2516, and LQC 2520 of *L. plantarum* subsp. *argentoratensis* have been isolated from spontaneously fermented Greek wheat sourdoughs [8]. Information on the whole-genome sequencing of the strains and the subsequent bioinformatics processing of the raw reads (fastq files), up to genome assembly and annotation, can be found in the work of Syrokou et al. (2021) [9] as well as in the NCBI database (GenBank) under the accession numbers JAEQMR01, JAEQMQ01, JAEQMP01, JAEQMO01, JAEQMN01, and JAEQMM01, respectively.

### 3.2. Strains Relatedness

Overall genome-related index (OGRI) analysis [48] was performed according to Mataragas (2020) [49]. Species relatedness was examined by comparing the average nucleotide identity (ANI) and digital DNA-DNA hybridization (dDDH) values between the current strains and the type strains of *L. paraplantarum* DSM 10667^T^, *L. plantarum* subsp. *plantarum* ATCC 14917^T^, *L. plantarum* subsp. *argentoratensis* DSM 16365^T^, and *L. pentosus* DSM 20314^T^, downloaded from the GenBank database. The above phylogenetic neighbors were chosen following the workflow of genome-based classification at the species level proposed by Chun et al. (2018) [50]: first, the full length of the 16S rRNA gene sequence was extracted from the whole-genome sequences of each studied strain using the ContEst16S tool [51], and then the extracted sequences were loaded on the 16S-based ID platform of the EzBioCloud server [52] for identifying a bacterial isolate based on the 16S rRNA gene database of type strains. Bacterial identifications that displayed a high 16S sequence similarity value (≥98.7%) were further selected for OGRI analysis. The ANI values (threshold for species identification 95–96%) were calculated with the Orthologous Average Nucleotide Identity Tool (OAT) [53] while dDDH values (threshold for species and subspecies identification 70% and 79%, respectively) with the Type (Strain) Genome Server (TYGS) [54]. The TYGS was also used for establishing a bootstrapped genome-based phylogenetic relationship of the strains.

### 3.3. Phenotypic Characterization and Pan-Genome Analysis

Phenotype assignments to bacterial genomes of *L. plantarum* subsp. *argentoratensis* strains were achieved through the Traitar web service, accounting for 67 diverse traits [20]. Pan-genome analysis was conducted using the BPGA v1.3.0 software [55]. For the core-genome extraction, the default values of the tool were used; USEARCH software v10.0.240 [56] with a threshold of sequence identity equal to 0.5 (50%). The functional genes from the core-, accessory- and unique-genome of the *L. plantarum* subsp. *argentoratensis* strains were clustered to each COG and KEGG category with the USEARCH software, against the respective databases, embedded in the BPGA pipeline, applying the default parameters. Finally, the clusters of functional genes (pan-matrix), presented as a binary matrix (presence or absence), were introduced in the GENE-E software (https://software.broadinstitute.org/GENE-E/) (accessed on 6 September 2021) for visualizing the genes against the genomes. A hierarchical clustering (HC) of genes and genomes was performed using the option one minus the Pearson correlation distances available in the GENE-E. The web services PATRIC v3.6.8 [57], KofamKOALA [58], and eggNOG-mapper [59] were used to annotate the predicted proteins obtained during the pan-genome analysis of *L. plantarum* subsp. *argentoratensis* strains. The results of eggNOG-mapper also offered CAZymes analysis towards the CAZymes database [60].

### 3.4. Genomic Aspects Related to Food Safety

A safety assessment of the *L. plantarum* subsp. *argentoratensis* strains was performed by using whole-genome analysis and following the guidelines and recommendations of Chokesajjawatee et al. (2020) [6] and the EFSA FEEDAP Panel (2018) [10], respectively.

Antibiotic resistance and potential pathogenicity of the microbes were checked with the Resistance Gene Identifier (RGI) tool of the CARD [61] and PathogenFinder [62], respectively, following the analytical approaches of Rodrigo-Torres et al. (2019) [16]. Moreover, the egg-NOG mapper results based on the KEGG database was searched for undesirable genes as suggested by Chokesajjawatee et al. (2020) [6]. Bacteriocin-encoding genes were inspected using the BAGEL4 webtool [63]. Annotation results from PATRIC v3.6.8, KofamKOALA and eggNOG-mapper were screened for the ability of the strains to produce biogenic amines. Finally, the stability of the genomes was evaluated by investigating the following genetic features: (a) plasmids and insertion sequences (IS) were analyzed by PlasmidFinder [64] and MobileElementFinder [65], respectively. MobileElementFinder was also able to annotate acquired antimicrobial resistance genes using ResFinder [66,67] and virulence genes using VirulenceFinder [68]; (b) regions of Clustered Palindromic Interspaced Palindromic Repeats (CRISPR) with *cas* genes were detected with the CRISPRCasFinder webserver [42] using the default parameters; and (c) the presence of prophages was searched by means of the PHASTER webtool [69]. According to Chokesajjawatee et al. (2020) [6], the WHO CIA list (list of critically important antimicrobials of World Health Organization) served as a reference to evaluate the ability of the microbial strains to produce antimicrobial drugs of clinical importance via the KofamKOALA and egg-NOG-mapper results of the KEGG database.

## 4. Conclusions

This work deals with the comparative genomic analysis of six *L. plantarum* subsp. *argentoratensis* strains previously isolated from Greek wheat sourdoughs. The analysis showed that the lactic acid bacteria possessed properties with biotechnological/probiotic potential (e.g., as a starter culture in fermented foods). Their safety based on the EFSA recommendations was evaluated. Regarding biogenic amines production, the six strains encoded a lysine carboxylase gene, *cadA*, associated with cadaverine production but the real risk of such production and accumulation should be confirmed phenotypically. No virulence factors or production of antimicrobial drugs were detected in any of the queried genomes. Genes related to antibiotic resistance were found but the experimental validation of this resistance was not performed. Our aim was the bioinformatic characterization of the *L. plantarum* subsp. *argentoratensis* strains and the evaluation of their safety to have a reference study for any future validation experiment involving the current bacteria. Similar resistance antibiotic genes, however, are frequently encountered in strains of the same species including probiotics (WCFS1, JDM1, ST-III, and 299V) [6]. All microorganisms were able to produce plantaricins belonging to different classes. Finally, plasmids, prophage regions, insertion sequences, and CRISPR*cas* systems were identified in the six genomes. Consequently, the in-silico analysis performed and the genomic results obtained guarantee the safety of the microbial strains for food applications. 

## Figures and Tables

**Table 1:** Pairwise comparisons (dDDH values) between the genomes of the strains isolated from traditional Greek wheat sourdoughs (LQC 2422, LQC 2441, LQC 2516, LQC 2485, LQC 2320, and LQC 2520) and the genomes of the type strains restricted to those displayed a very high 16S rRNA gene similarity (≥99.80%).

a/a	Strains	1	2	3	4	5	6	7	8	9	10
1	LQC 2422	-									
2	LQC 2441	**99.9**	-								
3	LQC 2516	**100**	**100**	-							
4	LQC 2485	**99.5**	**99.5**	**99.5**	-						
5	LQC 2320	**89.2**	**89.2**	**89.2**	**88.9**	-					
6	LQC 2520	**89.2**	**89.2**	**89.2**	**88.9**	**100**	-				
7	*L. paraplantarum* DSM 10667^T^	31.1	31.2	31.2	31.5	31.1	31.2	-			
8	*L. plantarum* subsp. *argentoratensis* DSM 16365^T^	**95.1**	**95.1**	**95.2**	**94**	**89.9**	**90.5**	31.5	-		
9	*L. plantarum* subsp. *plantarum* ATCC 14917^T^	62.5	62.4	62.4	62.3	63.1	63	31.1	62.8	-	
10	*L. pentosus* DSM 20314^T^	24.4	24.4	24.4	24.5	24	24	24.4	24.9	23.8	-

Values in bold indicate that the threshold value for subspecies (79%) or species (70%) delineation is exceeded.

**Table 2:** AMR (antimicrobial resistance) genes identified in the *L. plantarum* subsp. *argentoratensis* genomes.

a/a	AMR Element	KEGG KO	Gene (EC No)	Description
1	Bacitracin	K06153	*uppP*, EC 3.6.1.27	Catalyzes the dephosphorylation of the undecaprenyl diphosphate (UPP). Confers resistance to bacitracin
				ABC transporter, permease protein, probably the 2 or 3 component bacitracin resistance efflux pump, BcrAB or BcrABC
				Chloramphenicol acetyltransferase
2	Bacitracin	K01992	-	D-alanyl-D-alanine-carboxypeptidase
				Catalyzes hydrolysis of the D-alanyl-D-alanine dipeptide
				Beta-lactamase enzyme family
3	Phenicol	K19271	*catA*, EC 2.3.1.28	Mediates bacterial resistance to the antibiotics streptomycin and spectomycin
4	Vancomycin	K07260	*vanY*, EC 3.4.17.14	
5	Vancomycin	K08641	*ddpX*, EC 3.4.13.22	
6	Beta-Lactam	K17836	*bla1*, *bla2*, EC 3.5.2.6	
7	Aminoglycoside	K00984	*aadA*, EC 2.7.7.47	

**Table 3:** CRISPR*cas* system identified in the *L. plantarum* subsp. *argentoratensis* genomes.

Strain	Total CRISPR Regions	CRISPR Regions with EL 1 or 2 ^1^	CRISPR Regions with EL 3 or 4	*cas* Gene Clusters Nearby CRISPR Regions ^2^	Type of *cas* Gene Cluster	Total *cas* Genes ^3^
LQC 2320	4	2 of EL1	2 of EL4	2	CAS-typeI and CAS-typeIE	11
LQC 2422	4	1 of EL1	1 of EL3 & 2 of EL4	2	CAS-typeI and CAS-typeIE	11
LQC 2441	4	1 of EL1	1 of EL3 & 2 of EL4	2	CAS-typeI and CAS-typeIE	11
LQC 2485	5	1 of EL1	4 of EL4	2	CAS-typeI and CAS-typeIE	11
LQC 2516	4	1 of EL1	1 of EL3 & 2 of EL4	2	CAS-typeI and CAS-typeIE	11
LQC 2520	4	2 of EL1	2 of EL4	2	CAS-typeI and CAS-typeIE	11

^1^ When the evidence level (EL) is 1 or 2 then the CRISPR region is not likely to be a real CRISPR array. ^2^ Only two CRISPR regions of evidence level (EL) 4 had in vicinity *cas* gene clusters (one gene cluster per CRISPR array). ^3^ CAS-typeI gene cluster, four *cas* genes; CAS-typeIE, seven *cas* genes.

## Data Availability

The genome sequences are available in the NCBI Bioproject ID PRJNA689714 [9].
